# Draft Genome Sequence of Clostridium bowmanii DSM 14206^T^, Isolated from an Antarctic Microbial Mat

**DOI:** 10.1128/MRA.01035-21

**Published:** 2022-01-06

**Authors:** Faith P. Palevich, Nikola Palevich, Paul H. Maclean, Eric Altermann, John Mills, Gale Brightwell

**Affiliations:** a AgResearch Ltd., Hopkirk Research Institute, Palmerston North, New Zealand; b AgResearch Ltd., Grasslands Research Centre, Palmerston North, New Zealand; c Riddet Institute, Massey University, Palmerston North, New Zealand; d New Zealand Food Safety Science and Research Centre, Massey University, Palmerston North, New Zealand; University of Rochester School of Medicine and Dentistry

## Abstract

Clostridium bowmanii type strain DSM 14206 (ATCC BAA-581) was isolated from a microbial mat sample retrieved from Lake Fryxell, Antarctica. This report describes the generation and annotation of the 4.9-Mb draft genome sequence of C. bowmanii DSM 14206^T^.

## ANNOUNCEMENT

Clostridium bowmanii DSM 14206^T^ (ATCC BAA-581^T^) is a Gram-positive, spore-forming, and slow-growing psychophilic anaerobe with an optimum temperature range of 12°C to 16°C (1) that was originally isolated from a mat sample from the shallows surrounding a moat on the Antarctic Lake Fryxell (2). C. bowmanii has been identified as a spoilage microorganism in chilled meat products and vacuum-packed meat without the production of gas. While initial phylogenetic analysis based on 16S rRNA gene sequence data placed DSM 14206^T^ closest to the clostridial cluster containing Clostridium estertheticum type strain DSM 8809 (ATCC 51377) (2), subsequent 16S rRNA gene and phylogenomic analyses placed DSM 14206^T^ closest to Clostridium tagluense DSM 17763^T^ (3) and *Clostridium* sp. strain FP3, which was cultivated from a spoiled chilled vacuum pack of lamb meat (4–6).

Strain DSM 14206^T^ was acquired from the Leibniz Institute DSMZ (German Collection of Microorganisms and Cell Cultures) and cultured anaerobically at 12°C in prereduced peptone-yeast extract-glucose-starch (PYGS) broth (7). Genomic DNA was extracted using a modified phenol-chloroform procedure (8) and was mechanically sheared using a nebulizer instrument (Invitrogen) to select fragments of approximately 550 bp. A DNA library was prepared using the Illumina TruSeq Nano method and sequenced on the Illumina MiSeq platform with the 2 × 250-bp paired-end (PE) reagent kit v2, producing a total of 4,394,200 PE raw reads. The A5-miseq pipeline v20169825 with standard parameters was used to check the quality of the raw reads and subsequent read trimming and assembly (9). In addition, the *de novo* assembly of the MiSeq reads was merged with the publicly available DSM 14206^T^ assembly (GenBank assembly accession number ASM1886131v1) using Quickmerge v0.3 (10). The trimmed Illumina reads were mapped back to the refined assembly using BWA v0.7.17-r1188 (11), and contigs to which no unique reads mapped were removed.

The reported and improved assembly consists of 92 contigs, with 202× coverage and an *N*_50_ value of 134,761 bp; the largest scaffold is 280,085 bp in length. The draft genome sequence is composed of 4,882,709 bp, with a G+C content of 31.2%. A total of 4,546 putative protein-coding genes (PCGs), along with 88 tRNA and 16 rRNA elements, were predicted using Prokka v1.14.5 (12) and GAMOLA2 (13). All bioinformatic analyses were performed using default settings and parameters.

Overall, we produced an improved draft genome sequence of DSM 14206^T^, with increased sequence coverage and quality of genomic information but with a significant reduction in the number of contigs, by combining Illumina sequence technologies. Additional annotation via carbohydrate-active enzyme profiling using dbCAN2 (14) revealed that the DSM 14206^T^ genome encodes 54 glycoside hydrolases, 53 glycosyl transferases, 1 polysaccharide lyase, 20 carbohydrate esterases, and 7 carbohydrate-binding protein module families. Clustered regularly interspaced short palindromic repeats (CRISPRs) were identified, with a large 7.7-kb CRISPR1-Cas with flanking *cas* genes on scaffold 35. The draft DSM 14206^T^ genome sequence reported here is a valuable resource for future studies investigating psychrophilic *Clostridium* species and their spoilage properties.

### Data availability.

The genome sequence and associated data for Clostridium bowmanii type strain DSM 14206 (ATCC BAA-581) were deposited under GenBank accession number JAIRAZ000000000, BioProject accession number PRJNA574489, and Sequence Read Archive (SRA) accession number SRR15734597.
